# Companion diagnostics: new opportunities for safe and effective anti-infectious disease therapies

**DOI:** 10.1038/s41426-017-0014-9

**Published:** 2018-01-24

**Authors:** Yong-Jie Zhou, Guanghua Wang, Yi-Wei Tang

**Affiliations:** 1 Office of Tissues and Advanced Therapies, Center for Biologics Evaluation and Research, Food and Drug Administration, Silver Spring, MD 20993 USA; 2 Division of Molecular Pathology, Joint Pathology Center, Bethesda, MD 20889 USA; 3 Department of Laboratory Medicine, Memorial Sloan-Kettering Cancer Center, Department of Pathology and Laboratory Medicine, Weill Medical College of Cornell University, NewYork, NY 10065 USA

Infectious diseases remain a tremendous burden for the public health and economic growth worldwide^1^. Emerging and re-emerging microbes, antimicrobial resistance, therapeutic product-induced toxic effects, and variations in efficacy for anti-infectious disease therapies consistently create challenges in controlling infectious diseases^1–3^. Notably, current available medical products are not sufficient to prevent or cure patients in many infectious diseases^1,2^. Thus, development of effective medical products for diagnosis, treatment, and prevention of infectious diseases is greatly needed. Co-development of companion diagnostic devices (CDxs) and their corresponding therapeutic products (CDxs/therapeutics) provides new opportunities for safer and more effective anti-infectious disease therapies^4^.

A CDx is a medical device, often an in vitro diagnostic device (IVD) or an imaging tool, which provides information essential for the safe and effective use of a corresponding therapeutic, preventive, or prophylactic product as defined by the U.S. Food and Drug Administration (FDA)^4–6^. The use of a CDx is stipulated in the instructions in FDA-approved labeling for both the CDx and the corresponding therapeutic product. A CDx test can help health care providers determine whether the patients can receive the corresponding therapeutic product before it is prescribed. However, an IVD is not considered to be a CDx if the indicated IVD does not provide information essential for the safe and effective use of a corresponding therapeutic product^6^. In most circumstances, a CDx and its corresponding therapeutic product are approved or cleared contemporaneously by FDA for the use indicated in the therapeutic product labeling.

Co-development of a CDx for a safer and more effective use of the corresponding therapeutic product can significantly protect and promote the public health^4–7^. A CDx can be used to select the patients who are most likely to benefit, or to be at an increased risk for serious therapeutic product-induced toxicities following administration of a specific therapeutic product. A CDx can also be used to monitor the patients’ responses to a specific therapeutic product for the purposes of adjusting an optimal dosing regimen for a safer and more effective use of a specific therapeutic product^4–6^. When a CDx is used to select a subpopulation of patients for a clinical trial to establish the safety and effectiveness of a specific therapeutic product, product developers may reduce the costs and shorten the time to achieve an approval by FDA for the corresponding therapeutic product^7^.

Successes in CDx/therapeutic co-development are mostly achieved in the field of anti-cancer targeted therapies. The first co-developed CDx/therapeutic product, a semi-quantitative immunohistochemistry assay HERcep Test and its therapeutic product HERCEPTIN, was approved by FDA in 1998 for the treatment of breast cancer. Since then, FDA has cleared or approved more than 30 CDxs in the field of oncology^4,6^. Based on the analysis of therapeutic products with CDxs that are on the market, oncology may remain to be the main area for the CDx/therapeutic co-development in the foreseeing future. However, other fields are beginning to emerge for the CDx/therapeutic co-development, including infectious diseases^7^.

Currently, medical treatments in the field of infectious diseases are generally designed for the “average patient” as “one-size-fits-all-approach”, which is safe and effective for some patients, but not for others^8^. Precision medicine provides a great potential to improve prevention and treatment for human diseases, including infectious diseases, by taking individual variability into account, often defined at the molecular and cellular levels^8,9^. Co-development of CDx/therapeutic products gives a promise to deliver the next generation medicine of anti-infectious diseases (i.e., the precision medicine) to the right patients at right dose regimen^4,8–10^. Human immunodeficiency virus type 1 (HIV-1) tropism testing is an example of CDxs in the field of infectious diseases^11^. The HIV-1 tropism testing can identify patients who are only infected with CCR5-tropic HIV-1 for the effective use of antiviral drug maraviroc. Maraviroc specifically inhibits the entry of CCR5-tropic HIV-1 to the host cells, but does not inhibit the entries of CXCR4-tropic and dual/mixed-tropic HIV-1 to the host cells. Based on the clinical trial data, the FDA-approved-label for maraviroc requires that the HIV-1 tropism testing be conducted before it is prescribed^11,12^.

Many host variations identified in human genomic, pharmarcogenomic, and proteomic studies play important roles in pathogenesis and prognosis of infectious diseases, and the safety and effectiveness of the therapeutic products^3,8,13^. These scientific discoveries provide great opportunities to pharmaceutical and diagnostic industries, academic institutions, and medical care facilities to develop the next generation medicine to combat infectious diseases. For examples, genome-wide association studies found that HLA-B*5701 allele is associated with severe hypersensitivity reactions to abacavir and CYP2B6*/*6 alleles is associated with the central nerve system disorders to efavirenz in patients with HIV-1 infection^3,13^. A variant in IL-28B gene is associated with a poor response to pegylated interferon alfa and ribavirinin patients with chronic hepatitis C virus infection^3,13^. Additionally, expression of programmed death (PD)-1 protein on CD8T-cells is associated with T-cell exhaustion and disease progression in patients with HIV-1 infection. Blockade of the PD-1/PD-ligand 1 pathway restores CD8T-cell function and reduces viral load, indicating a potential option to pursue for co-development of CDx/therapeutic products to improve anti-HIV therapies^14^.

With further advancement in science, medicine, and technology, successful co-development of CDx/therapeutic products will deliver more precision medicines for diagnosis, treatment, and prevention of infectious diseases and reduce the public health burdens.

## Disclaimer

(1) This article reflects the views of the author and should not be construed to represent FDA’s views or policies. (2) The identification of specific products, scientific instrumentation, or organization is considered an integral part of the scientific endeavor and does not constitute endorsement or implied endorsement on the part of the author, DoD, or any component agency. The views expressed in this article are those of the author and do not reflect the official policy of the Department of Army/Navy/Air Force, Department of Defense, or U.S. Government.
